# The Trade-Offs between Optimality and Feasibility in Online Routing with Dedicated Path Protection in Elastic Optical Networks

**DOI:** 10.3390/e24070891

**Published:** 2022-06-29

**Authors:** Ireneusz Olszewski, Ireneusz Szcześniak

**Affiliations:** 1 Faculty of Telecommunications, Computer Science and Electrical Engineering, Bydgoszcz University of Science and Technology, S. Kaliskiego 7, 75-796 Bydgoszcz, Poland; 2 Department of Computer Science, Częstochowa University of Technology, 42-200 Częstochowa, Poland; ireneusz.szczesniak@pcz.pl

**Keywords:** elastic optical networks, dedicated backup path protection, branch and bound method

## Abstract

The article discusses an online problem of routing and spectrum allocation with dedicated path protection in elastic optical networks. We propose three novel algorithms to solve this problem. The first of them is the minimum-cost–maximum-flow heuristic algorithm, which calculates the solution assuming that the spectrum units on the working and dedicated backup path are the same. Such an assumption, on the one hand, increases the bandwidth blocking probability; however, on the other hand, it enables a simple, cheap and fast way to connect customers to the network during the implementation phase of elastic optical networks. The next two algorithms, which determine the exact solutions, are based on the branch and bound method. The first calculates the working and dedicated backup paths with the minimum total occupied bandwidth, called the total cost, while the second calculates the paths with the minimum total length. These algorithms enable the performance evaluation of the proposed heuristic algorithm and provide the answer as to what should be optimized, the total cost or the total length of paths, in order to minimize the bandwidth blocking probability. Extensive simulation research has shown that the proposed heuristic algorithm can be used in elastic optical networks, but with a small network load. Moreover, it is shown that the optimization of the total cost of paths provides a slightly lower blocking probability than the optimization of the total length of paths.

## 1. Introduction

The spread of services on the telecommunications market, such as high definition television (HDTV), video on demand (VoD), cloud computing services, e-learning and fast Internet requiring a large bandwidth, makes it necessary to transfer huge volumes of traffic in IP networks. It was estimated that from 2017 to 2022, the value of Internet traffic should increase by 26% per year [1]. Therefore, network operators expect reliable optical transport networks capable of handling such large traffic. The response to such expectations of operators are elastic optical networks (EON) based on orthogonal frequency division multiplexing (OFDM). This multiplexing offers the possibility of flexible transmission of signals in the network with very different bit rates, in the order of Gb/s, on specified spectrum units, which allows the avoidance of energy-consuming traffic aggregation or channel bonding. The use of OFDM ensures a significant improvement of network utilization compared to the dense wavelength division multiplexing network (DWDM). It is obvious that the failure of even a single link in such a network can cause serious disruptions in the transmission of traffic for many customers. The fault resistance of links in EON network can be increased, as in the WDM network, using protection or restoration mechanisms. The solution for network protection is to use a dedicated or shared backup path protection for each working path.

It should be expected that the technology of elastic optical networks will be implemented gradually, through evolution, similarly to WDM technology. Therefore, one should not expect that each node of future elastic optical networks will be immediately equipped with an elastic add-drop multiplexer (EADM) or bandwidth-variable cross-connect (BV-WXC) [2]. One should expect, especially at the beginning of implementation, simple and cheap solutions that meet the functional and financial requirements of network operators. One of the popular devices in current WDM networks is the fiber protection unit [3], which allows the user to set up a connection with a dedicated backup path cheaply by introducing the wavelength generated directly by the customer into the operator’s network. It can be expected that similar devices will find application in EON. A customer on both sides of the connection would generate a signal on the given FSUs using demarcation equipment. Fiber protection units can be located on the customer’s side, if the customer wants to be connected to the operator’s network with two fibers. Before entering the signal to the network, the operator would have to apply filters for the used FSUs to protect against possible attacks. The signal with the required FSUs can also be generated in the operator’s station using a single EON transceiver, without the need to use an EADM or a BV-WXC.

This article focuses on the online RSA problem with dedicated path protection (DPP) in EON. Because such a problem cannot be solved tractably [4], even for small networks, it is necessary to propose heuristic algorithms. A proposed algorithm solves this problem by assuming that the FSU indexes on the working and dedicated backup path are the same. This assumption will be very important during the implementation phase of EON, enabling the easy and fast connection of the customer to the network. To verify the proposed algorithm, two exact algorithms based on branch and bound method were proposed. The first optimizes the amount of bandwidth occupied by the request; the second one the total length of the paths. These algorithms also answer the question as to what should be optimized—occupied bandwidth or the total length of paths—in order to minimize the bandwidth blocking probability in the network.

The novel contributions of this article are the heuristic algorithm determining the approximate solution and the two algorithms that determine the exact solutions for the RSA problem with DPP. In addition, results obtained during extensive simulations are also a contribution of this article.

The essence of this article is not only to show the results of research obtained based on the proposed algorithms, but also to show the obtained trade-offs in the case of connections with a DPP in the proposed way. On one hand, it is possible to obtain a cheap and fast solution that is easily implemented; on the other hand, to obtain a much higher bandwidth blocking probability compared to optimal solutions, by using less efficient modulation on the working path, if such a modulation is required on the (longer) dedicated backup path.

The further plan of the article is as follows. In the second part, the related works are shown. In the third part, the problem statement is presented. In the fourth part, the heuristic algorithm is proposed, while in the fifth part, the algorithms based on the branch and bound method are shown. The sixth part presents the results obtained for example networks. The seventh part contains a summary and conclusions.

## 2. Related Works

In the literature, many articles can be found concerning the offline routing and spectrum allocation problem (RSA) with dedicated or shared path protection [5,6,7,8,9,10]. However, there are definitely fewer articles on the online RSA problem with dedicated or shared path protection.

The online RSA problem, in contrast to the offline RSA problem, is characterized by the fact that the requests arrive randomly (they are not known in advance), for example according to the Poisson process with exponential service time. However, in the case of an offline problem, the set of all requests is known in advance. Requests can be allocated simultaneously or one by one at any time and are not disconnected.

In [11], an online RSA problem with shared path protection (SPP) was used to increase the usage of the spectrum compared to dedicated path protection. In this protection, two backup paths, calculated for two link-disjoint working paths, share the backup spectrum on the link adjacent to these paths.

In [12], an online RSA problem with SPP in EON is considered. The algorithm solving this problem is based on spectrum window planes (SWPs), with the weight of each SWP edge equal to the required number of frequency slot units (FSUs) for the request. The working path is the shortest path, measured by the number of links (in hops) from the paths calculated for all SWPs. The shared backup path is determined analogously on the basis of SWPs; however, the weight of each SWP edge depends on the number of SPPs that share the slots of this edge. The main disadvantage of this algorithm is the long calculation time associated with a large number of SWPs for the request.

In [13], the online RSA problem with SPP is solved on the basis of the Primary First-Fit Modified Backup Last-Fit algorithm. The primary path in this algorithm is selected from the set of k-shortest paths sorted in ascending order by length. The spectrum allocation is based on the First-Fit (F-F) spectrum allocation policy. The shared backup path is also selected from the set of k-shortest paths calculated for the graph from which the primary path was temporarily removed. From the k-shortest paths, the shared backup path with the smallest penalty function is selected using Last-Fit (L-F) spectrum allocation policy. The penalty function depends on the position of the solution with respect to the end of the spectrum (in FSUs) and the number of requested FSUs.This algorithm was used to analyze the obtained results.

In [4], the modulation-adaptive link-disjoint path selection problem is formulated as an integer linear programming (ILP) to minimize the number of required FSUs for link-disjoint paths between a pair of nodes in EONs. In this paper it has been proven that modulation-adaptive link-disjoint path selection problem is NP-complete.

## 3. Problem Statement

Let us assume that such a network is given as a graph G(N,E), where *N* is a set of nodes and *E* is a set of unidirectional links. The network is equipped with bandwidth-variable transponders, which can support various modulation formats, e.g., BPSK, QPSK, 8-QAM, 16-QAM and bandwidth-variable wavelength cross-connect [2]. Let *D* be the set of link lengths $d_{i,j}$, i,j∈N and $F=F_{1},F_{2},\ldots ,F_{\left|F\right|}$ denote the set of FSUs supported by the transmission system on each network link, where $F_{i}$ corresponds to the *i*-th FSU. In addition, *g* is the guard band between adjacent connections on the network links. In turn, the triple (s,d,C) determines the request *C* Gb/s from node *s* to node *d*.

The solution of the online RSA problem with DPP for request *C* Gb/s between the pair of nodes *s*, *d* contains two link-disjoint paths, the working path and dedicated backup path, which optimize the adopted objective function. Each of the paths must meet the spectrum continuity and contiguity constraints, and non-overlapping spectrum constraint for adjacent requests on links of each path. Meeting the spectrum continuity constraint requires FSUs with the same indexes on all path links, while meeting the spectrum contiguity constraint requires FSUs with subsequent indexes on all links of this path. In the case of a link failure on the working path, the traffic of *C* Gb/s is received from the backup path.

The article discusses the dynamic RSA problem with DPP for two different objective functions. In the first of them, the total amount of occupied bandwidth on both paths is being minimized, while in the second, the total length of paths is being minimized. The bandwidth for each path, called the cost, is defined here as the product of the path length and the required number of FSUs, while the path length is the sum of the link lengths belonging to the path. The required number of FSUs *n* for request is calculated on the basis of Equation (1) (Table 1 in [7,14]).

(1) n=⌈C/12.5m⌉+g

where: *m* is the modulation level for a given modulation format.

The denominator of this expression (12.5 m) specifies the part of traffic, expressed in Gb/s, transferred by a single FSU for a given modulation format for which m∈1..M, where *M* is the highest modulation level (Table 1). It should be noted that the calculated paths can be of different lengths and hence may require different modulation. To minimize total length or the total cost of both paths, the obtained solution must maximize the modulation level on each path.

In order to justify the consideration of the problem for two different objective functions, the following example will be presented. Let us assume that for the realization of a request on distances up to 250 km, the applied modulation format requires one FSU, while on distances more than 250 km requires two FSUs. It is assumed here that after a reach of 250 km, a less spectrally efficient modulation format should be used; however, the consequence of this is a larger number of slots.

Consider two cases: in the first of them there are two separate paths of 250 km each. Thus, the total length will be 500 km. In the second case, there are also two paths of the same total length, however the length of the first one is equal to 200 km and the second one is equal to 300 km. Both in the first and second case, the algorithm optimizing the total length of paths will calculate the same solution equal to 500 km, while the algorithm optimizing the total cost of paths will calculate two different solutions. In the first case, the total cost will be 250 × 1 + 250 × 1 = 500, while in the second one it will be 200 × 1 + 300 × 2 = 800. This simple example shows that the solutions can be different for different objective functions in the problem under consideration.

In this article three algorithms have been proposed to solve RSA problem with DPP. The first one is a heuristic algorithm, while the next two are exact algorithms based on the branch and bound method.

The heuristic algorithm, called the Minimum Cost Maximum Flow (MCMF), optimizes the total cost of paths in the considered problem. Paths are calculated assuming that the required number of FSUs with the same indexes is determined on both paths, regardless of path lengths. For paths of different lengths, the required number of FSUs on the longer path is assumed. Adoption of this assumption would significantly facilitate in the implementation of the elastic optical network.

In order to verify the heuristic algorithm, two exact algorithms based on the branch and bound (BB) method were proposed. The first of them, the BB_Cost algorithm optimizes the total cost of both paths and the second of them, the BB_Length algorithm optimizes the total length of both paths. The proposed BB algorithms will explain which object function should be selected in the problem under consideration to minimize the bandwidth blocking probability in the network.

## 4. Heuristic Algorithm

The proposed heuristic MCMF algorithm uses the minimum-cost–maximum-flow algorithm [15] to find a pair of paths in filtered graphs trying to minimize the total cost of paths. In a filtered graph, only those links are kept that can support a given slot. A slot is a sequence of specific contiguous FSUs, i.e., a slot of unit indexes from 0 to 20 is different from a slot of unit indexes from 20 to 30. For a given request, we create a list of all slots that can support it, and sort the list in the ascending order of the number of FSUs the slots have.

Next, we iterate over the slots in batches of the equal number of required FSUs. For every slot in a batch, we search for a pair of paths using a minimum-cost–maximum-flow algorithm in a graph filtered for the given slot with the link capacities set to one. The minimum-cost–maximum-flow algorithm can find a single path, which is no solution for us, or more than two paths, in which case we choose two paths of the smallest cost, provided the length of each of the two paths is not larger than the reach of the modulation used for the slot. From the solutions found for the batch we iterate over, we select the pair of paths of the smallest cost, and return it with the given slot as the solution found. If no solution was found for the batch, we iterate over the next batch of slots in the list until there are no more batches to process.

The proposed algorithm is heuristic for two reasons. First, since both paths use the same slot, the algorithm cannot be optimal, because there can be paths of smaller cost which use different slots on the paths. Second, while it is a common technique to use a minimum-cost–maximum-flow algorithm to find a maximum number of paths of minimum-cost in a graph, we are not sure that two paths chosen from a larger number of paths will really be of minimal cost.

The pseudo-code of the MCMF algorithm is shown in Algorithm 1. *S* denotes a slot (a sequence of contiguous FSUs), and the function Ω(C,m) produces a sequence of slots (where slots of lower indecies go first) which can accommodate the signal of bitrate *C* Gb/s using modulation level *m*. The function SSPA(G(S),s,d) finds the minimum-cost maximum-flow from *s* to *d* in the filtered-graph G(S), and returns it as a list of paths sorted in the ascending order of the path cost. A filtered-graph G(S) is a subgraph of a graph G(N,E). Each link of G(S) has slot *S* available. SSPA is an acronym for the name of the minimum-cost–maximum-flow algorithm we used: the Successive Shortest Path Algorithm. The function count (Paths) returns the number of paths in the list Paths, $Path_{i}$ is the *i*-th path in Paths, and the function len($Path_{i}$) returns the length of the path $Path_{i}$. The reach of the modulation *m* is denoted as $r_{m}$.
**Algorithm 1** MCMF Algorithm {Approximate solution} Solutions=⌀; **for**
m=M to 1 **do**  **for all**
S⊂Ω(C,m)
**do**      Paths=SSPA(G(S),s,d);      **if** count(Paths)≥2  **then**          **if**  $len\left(Path_{1}\right)\leq r_{m}\;and\;len\left(Path_{2}\right)\leq r_{m}$  **then**                $Solutions=Solutions\cup \{Path_{1},Path_{2}\}$;          **end if**      **end if** **end for** **if**
Solution≠⌀**then**
     
**return** the cheapest solution and *S*;  **end if** **end for** **return** without a solution

The time and memory complexity of the heuristic algorithm depends on the minimum-cost–maximum-flow algorithm used. We used the successive shortest path algorithm [15], which is of time complexity $O(N_{d}\left(|E\right|+\left|N|\times log|N|)\right)$, where $N_{d}$ is the average node degree, and O(|E|+|N|log|N|) is the complexity of the Dijkstra algorithm. Since we run the successive shortest path algorithm at most $MW^{2}$ times, where *W* is the number of FSUs available in the network (the number of slots is (W+1)W/2), the time complexity of the proposed algorithm is $O(MW^{2}\times N_{d}\left(|E\right|+\left|N|log|N|)\right)$. The memory complexity is the same as the memory complexity of the successive shortest path algorithm (since we run it sequentially), which, in turn, is the complexity of the Dijkstra algorithm, i.e., O(|N|).

## 5. Exact Algorithms

The BB method is based on the analysis of the generated solution tree, which nodes represent all possible subsets of solutions for a given problem. The algorithm begins its operation in the tree root. Division of the tree root to the successors (children), which corresponds to the division of the whole set of solutions into separate subsets of solutions, follows the link of the path. Each subset of the solutions represented by a given successor of the tree root contains such solutions that have the first fixed link on the working path, coming out of the node *s*. Therefore, the number of subsets of solutions represented by the tree root successors is equal to the number of links leaving the node *s*.

Subsequent successors will be divided on the basis of the same principle until a solution is obtained. When splitting successive nodes of the solution tree, the links of the working path s−d are first established, and then the path s−d wraps to the node *s* and the links of the backup path d−s are established. Because the backup path is determined from the end node *d* to the begin node *s*, the links of this path must be selected for the opposite direction.

It should be noted that the number of nodes in the generated solution tree is an exponential function of the number of nodes of the graph *G*. The distance from the tree root to some solution in the generated solution tree, measured by the number of nodes, will be equal to the total number of nodes on both paths reduced by 1, because the node *d* occurs once and the node *s* occurs twice, once in the working path and once in the dedicated backup path.

For each node in the generated solution tree, a lower bound is calculated, which consists of two components: the first is the lower bound of the working path and the second is the lower bound of the dedicated backup path. The lower bound of each path is equal to the product of the total length of the fixed links in a given solution tree node and the required number of FSUs. The required number of FSUs is determined for a modulation format that supports transmission distance not less than the total length of fixed links of path. Of course, in the case where the fixed links in the considered tree node have not reached the destination node *d* yet, the lower bound of the backup path (second component) is equal to 0.

Browsing all the nodes of the generated solution tree, due to its exponential size, would be very time-consuming, therefore many nodes are closed. Closing of the node occurs when the lower bound of the node is greater than the cheapest solution found so far and when there is a loop on the working or dedicated backup path. Closing of the node of the solution tree will also occur when on the fixed links of both paths spectrum continuity and contiguity constraints are not met and when there are not two link-disjoint paths.

Shown below is the BB_Cost algorithm, which calculates the exact solution of the online RSA problem with DPP. The proposed algorithm searches the solution tree using the queue. In this algorithm, the solution tree is not generated explicitly. Each node in the tree represents a set of paths s−d−s, working path s−d and dedicated backup path d−s, in which some links have already been fixed. Let *T* be the node of the solution tree, where: T.WorkingPath is the path s−d, while T.BackupPath is the backup path d−s. The T.TotalCost=T.WorkingCost+T.BackupCost is the lower bound of the node *T*, where T.WorkingCost and T.BackupCost is the lower bound of the working and backup path, respectively. After fixing all the links on the path s−d and d−s, T.TotalCost will be the cost of one of the solutions.

**Figure 1:**
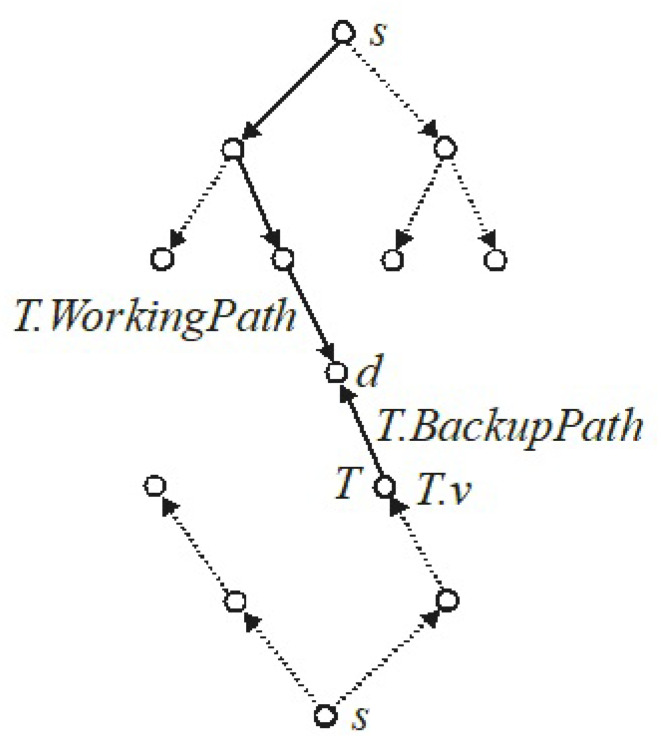
The solution tree generated by the BB_Cost algorithm.

Figure 1 shows the solution tree generated by the proposed algorithm. The selected node *T* of the solution tree contains the fixed links on the T.WorkingPath and the fixed first link on the T.BackupPath. Fixed links on both paths are marked with a solid line. T.v is a working node on the s−d−s paths.

Let the variables *X*, *Y* and TheBest contain the same fields as the variable *T*. In turn, AvailableUnits(Y.Path,n) is a function that for the aggregated bandwidth of the Y.Path searches, based on the First-Fit spectrum allocation policy, *n* available and contiguous FSUs. When there are no *n* of such FSUs, the AvailableUnits() is equal to false. The function len(Y.Path) returns the actual length of Y.Path. The IntoQueue() and FromQueue() functions write/read the node of the solution tree to/from the queue.

At first, the algorithm initializes the queue needed to search the solution tree by adding a tree root (line 6). Then, in the while loop, the nodes from the queue are read by FromQueue() (line 8). Each read node from queue is divided into its successors, which contain one more fixed link on the path than their parent.

At each node of the tree, it is checked whether the path being calculated s−d (Y.WorkingPath) does not contain a loop (line 14). Then, for the fixed links of the Y.WorkingPath, the maximum modulation level *m* is assigned (line 17) and the minimum number *n* of FSUs for the request with the *C* Gb/s is determined using Equation (1) (line 18). Next, spectrum aggregation of the fixed links of Y.WorkingPath is made and assigning the required *n* of FSUs by AvailableUnits function is done (line 19). Then, the lower bound, which is equal to the product of the length of Y.WorkingPath and required number *n* of FSUs, is calculated (line 24). After reaching the node *d* (line 20), the calculation of the working path is completed.

The way of determining the dedicated backup path is analogous to the working path; however, this path is calculated from the end node to the beginning node. It should be noted that the calculated modulation level on the backup path is generally different from that on the working path. These nodes in the generated solution tree, for which the lower bounds are smaller than the solution found so far, are written to the queue and further divided (line 61). After reaching the node *s*, it is compared whether the total cost of both calculated paths is lower than the TheBest.TotalCost found so far (line 56). The pseudocode of the BB_Cost algorithm is shown in Algorithm 2.
**Algorithm 2** BB_Cost Algorithm {Obtimal solution} TheBest.Weight=inf; T.Paths=s; T.WorkingPaths=s; T.v=s; IntoQueue(*T*); inicjalize Queue **while** Queue ≠ empty **do**    FromQueue(*X*);     **for all** children *u* of node X.v
**do**      Y=X;      StatusPath=false;      **if** Y.dWas=false **then**        {the node *d* has not been reached yet}        **if** u∉Y.WorkingPath **then**         {the loop does not appear on Working Path}         Y.WorkingPath=Y.WorkingPath+(Y.v,u);         m=m(Y.WorkingPath); calculation of the level of modulation         n=n(C,m); calculation of the required number of FSUs         **if** AvailableUnits(Y.WorkingPath,n) **then**           **if** u=d **then**              {the path s−d is calculated}              Y.dWas=true;          **end if**           Y.WorkingCost= len(Y.WorkingPath) × *n*;           StatusPath=true;         **else**           {closing the node- spectrum continuity and contiguity constraints are not met}         **end if**      **else**         {closing the node-the loop appear on Working Path}      **end if**    **else**      **if** u∉Y.BackupPath **then**         {the loop does not appear on the Backup Path}         **if** (u,Y.v)∉Y.WorkingPath **then**           {the paths are link-disconect}           Y.BackupPath=Y.BackupPath+(u,Y.v); adding a link in the opposite direction           m=m(Y.BackupPath); calculation of the level of modulation           n=n(C,m); calculation of the required number of FSUs           **if** AvailableUnits(Y.BackupPath,n) **then**              Y.BackupCost= len(Y.BackupPath) × *n*;              StatusPath=true;           **else**              {closing the node- spectrum continuity and contiguity constraints are not met}           **end if**         **else**           {closing the node-the paths are not link-disjoint}         **end if**      **else**         {closing the node-the loop appear on the Backup Path}      **end if**    **end if**    Y.v=u;    **if** StatusPath **then**      Y.TotalCost=Y.WorkingCost+Y.BackupCost;      **if** Y.TotalCost<TheBest.TotalCost **then**         **if** (u=s)and(Y.dWas) **then**           {the path d-s for opposite direction of the links are calculated}           TheBest=Y; a better solution has been found         **else**           IntoQueue(*Y*); the node should be divided         **end if**      **else**         {closing the tree node - only cheaper nodes than the solution found so far are considered}      **end if**    **end if**  **end for** **end while** **if** TheBest.TotalCost<inf 
**then**
    **return**
(TheBest.WorkingPath,TheBest.BackupPath) **else**    {request is blocked} **end if**

It should be noted that the order of the calculated paths in the proposed algorithm can also be reverse: first, the backup path and then the working path. The computational complexity function of the BB_Cost algorithm in the worst case is exponential.

The second algorithm, BB_Length, optimizes the total path length. This algorithm differs from the BB_Cost in that Y.WorkingCost is replaced by Y.WorkingLength, Y.BackupCost is replaced by Y.BackupLength, and Y.TotalCost is replaced by Y.TotalLength. In addition, the 24th line should be replaced by Y.WorkingLength = len(Y.WorkingPath); the 41st line should be replaced by Y.BackupLength = len(Y.BackupPath); the 55th line should be replaced by Y.TotalLength = Y.WorkingLength+Y.BackupLength; the 56th line should be replaced by **if** Y.TotalLength<TheBest.TotalLength **then**; and the 69th line should be replaced by **if** TheBest.TotalLength<inf **then**.

## 6. Obtained Results and Discussion

This chapter compares three algorithms: the heuristic MCMF algorithm and the two exact algorithms, the BB_Cost and the BB_Length, based on the BB method. Additionally, for comparative purposes, the results obtained on the basis of Primary F-F Modified Backup L-F [13] are presented. This algorithm was briefly shown in the introduction.

**Figure 2:**
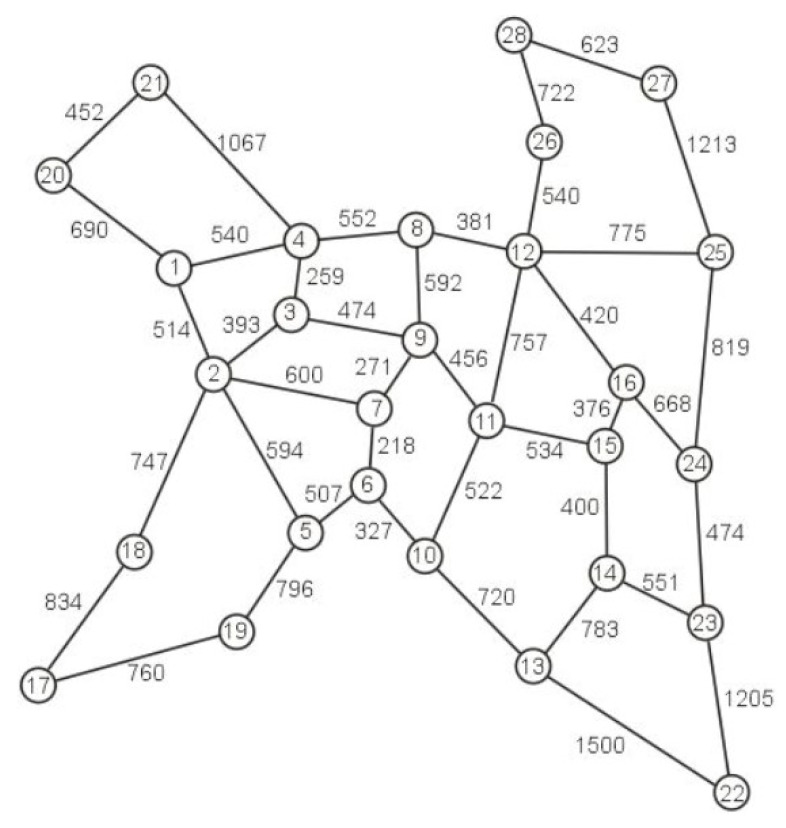
Network topology for Euro28.

**Figure 3:**
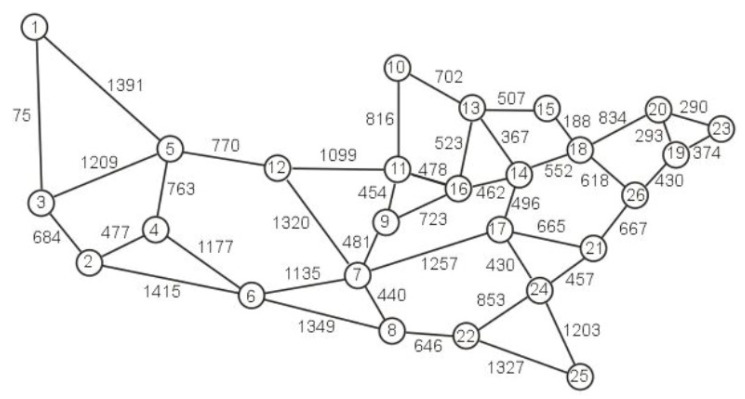
Network topology for US 26.

The research was carried out for two different networks. The first one is Euro 28 (Pan-European Nobel-EU network), which contains 28 nodes connected by 82 unidirectional links, while the second one is the US 26, which contains 26 nodes connected by 84 unidirectional links [14]. The topological structure of Euro 28 is shown in Figure 2 while the topological structure of US26 is shown in Figure 3. Each edge represents a pair of opposite directed unidirectional links and the numbers on the edges of the graphs of both networks determine the lengths of the links.

The transmission system on each network link supports 320 FSUs. Traffic offered to the network, in Erl, is defined as the sum of the durations of all incoming requests divided by the observation time. The traffic is affected by the intensity of the arriving requests and their durations. Thus, traffic intensity ρ can be expressed as the product of the number of requests arriving to the network per unit time λ and the average request duration 1/μ, hence ρ=λ/μ. The bit-rate *C* of each request is random from 20 to 200 Gb/s.

The investigation of all the proposed algorithms has been done by Monte Carlo simulation. For each incoming *C* Gb/s request between a pair of nodes, the given algorithm computes a link-disjoint pair of paths with the required number of FSUs, or it rejects the request if there is no bandwidth in the network. Based on the collected results, the number of rejected requests, bandwidth blocking probability, utilization of the network and average run time is determined depending on the network load.

The results are recorded when the system reaches a steady state, which occurs after the arrival of 1000 requests. The end of a single simulation run follows after the arrival of 50,000 requests. The estimation of simulation results for each network load was made on a limited 10-element set. For each result a 95% confidence interval was determined. Limiting the set to 10 for each load resulted from a very long calculation time for two exact algorithms: BB_Length and BB_Cost.

Table 1 gives the modulation formats, the distance of *l* (in km) for each of them and the bit-rate of the signal per each FSU [7,14].

**Figure 4:**
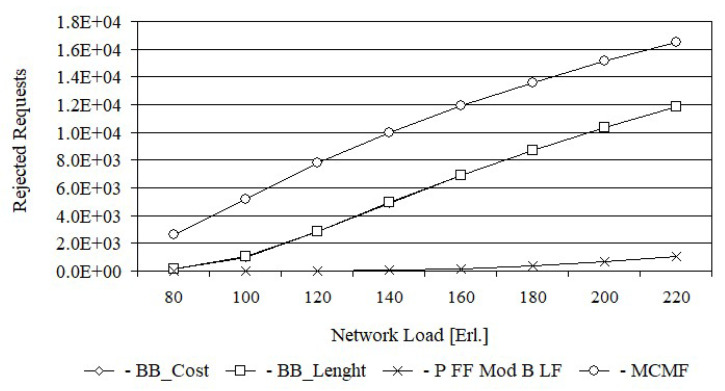
The number of rejected requests for Euro 28.

**Figure 5:**
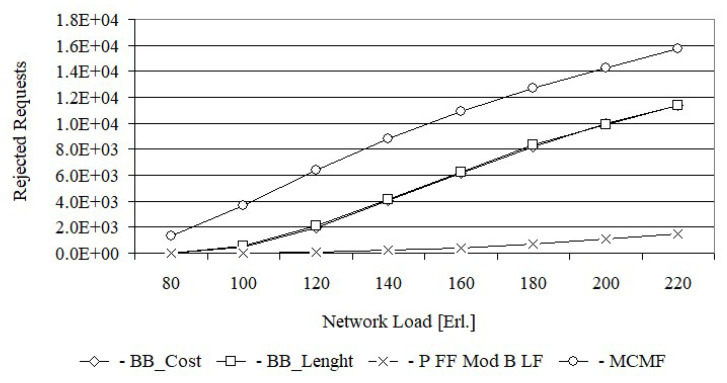
The number of rejected requests for US 26.

Figure 4 shows the number of rejected requests by each of the four algorithms in the Euro28 network, while Figure 5 shows the same characteristics for the US 26 network. Figure 4 shows that the lowest number of requests for each load is rejected by Primary FF Modified Backup LF; however, it should be noticed that this algorithm determines a working path with shared backup path.

The essence of this article is, among other things, a comparison of the proposed algorithms: MCMF with BB_Cost and BB_Cost with BB_Length. From Figure 4 it follows that above 100 Erl., the MCMF algorithm rejects around 4500 requests more than BB_Cost for each load. For smaller loads, however, the difference is much smaller. In turn, the number of rejected requests by the BB_Cost and BB_Length algorithms are almost the same for all network loads. However, when comparing specific values, it can be stated that for loads up to 180 Erl. slightly fewer requests are rejected by BB_Cost. For example, for a 140 Erl. load, BB_Length rejects 4915, while BB_Cost rejects 4864 requests. Analogical relationships between the numbers of rejected requests by the tested algorithms were obtained for the US 26 network.

Confidence intervals were determined for all loads, but the error bars were too small to plot. For example, for the 100 Erl. load, the number of rejected requests for the Euro28 network was 991.9 ± 24.0 for BB_Cost, 1026.6 ± 57.0 for BB_Length, 1 ± 0.1 for Primary FF Modified Backup LF and 5159.4 ± 101.0 for MCMF, while for the US26 network was 480.2 ± 55.7 for BB_CostB, 520.5 ± 59.3 for BB_Length, 8.5 ± 1.1 for Primary FF Modified Backup LF and 3670.5 ± 71.4 for MCMF.

**Figure 6:**
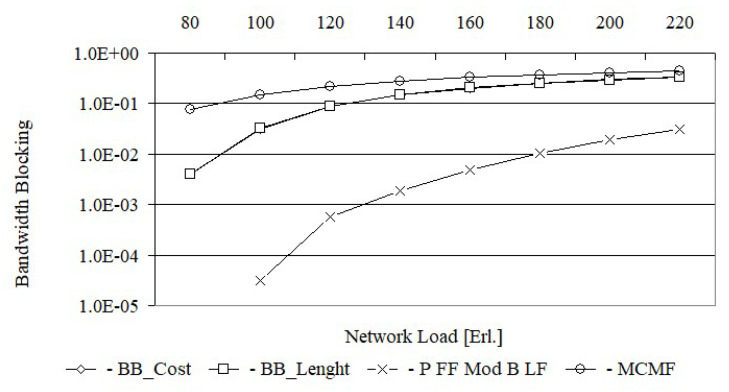
Bandwidth blocking probability for Euro 28.

**Figure 7:**
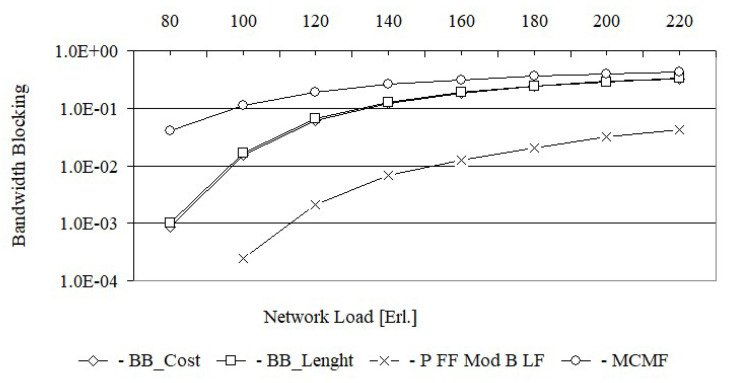
Bandwidth blocking probability for US 26.

In turn, Figure 6 shows the bandwidth blocking probability for the Euro 28 network after using each of the tested algorithms, while Figure 7 shows the same characteristics for the US26 network. The bandwidth blocking probability is defined here as the ratio of the rejected bandwidth to the offered bandwidth, i.e., $B=\sum_{i}{\bar{A}}_{i}C_{i}/\sum_{i}C_{i}$, where $A_{i}=1$ if the request is accepted for service by the network and $A_{i}=0$ if the request is rejected.

**Figure 8:**
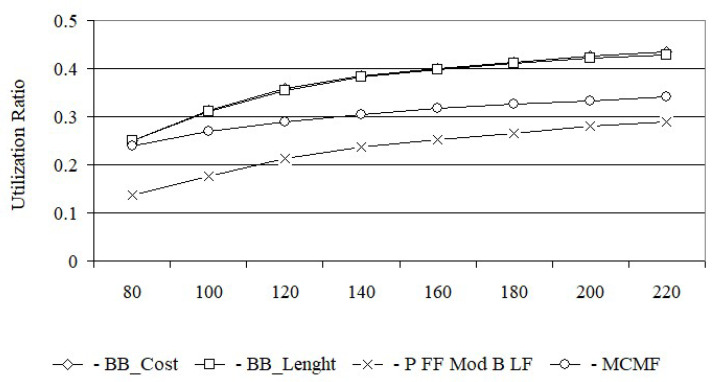
Utilization of the network for Euro 28.

**Figure 9:**
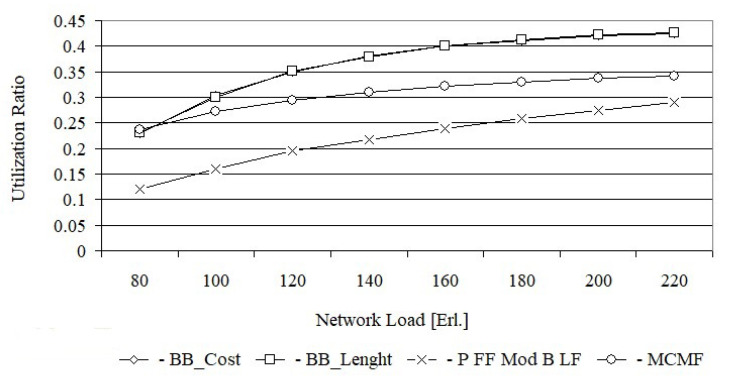
Utilization of the network for US 26.

From Figure 6, it follows that for a load equal to 80 Erl., the bandwidth blocking probability for the MCMF algorithm is still acceptable and equal to $7.8\times 10^{-2}$. However, for the BB_Cost and BB_Length algorithms, for the same load, the bandwidth blocking probabilities are much lower and equal to $3.9\times 10^{-3}$ i $4.1\times 10^{-3}$, respectively. With increasing load, e.g., up to 100 Erl., the bandwidth blocking probability for the MCMF algorithm is equal to $1.5\times 10^{-1}$ and is no longer acceptable. From this figure, it can be seen that for the first two loads the bandwidth blocking probability for the BB_Cost is slightly lower than for the BB_Length. For larger loads, the bandwidth blocking probabilities for both of these algorithms are the same. In turn, Figure 7 shows that the MCMF algorithm for a network load of 80 Erl. provides an acceptable bandwidth blocking probability equal to $4.0\times 10^{-2}$. However, it is almost two orders larger than the solution obtained on the basis of exact algorithms based on the BB method. It should be noted that for US 26, the bandwidth blocking probabilities for loads from 80 Erl. up to 180 Erl. are slightly smaller for the BB_Cost algorithm than for the BB_Length algorithm. In Figure 8 the spectrum utilization ratio for the Euro 28 network is shown, while in Figure 9 the spectrum utilization ratio for the US 26 network is shown. Spectrum utilization ratio is defined here as the average number of occupied FSUs on all network links divided by the total number of FSUs on the network links and is calculated for each of the 10 runs. The average number of occupied FSUs covers all changes of spectrum (connection and disconnection) in the network.

From Figure 8, it follows that for a network load equal to 80 Erl., spectrum utilization ratios for the BB_Cost, BB_Length and MCMF algorithms are comparable and equal to 0.25. As the network load increases, the spectrum utilization ratio for all tested algorithms increases too. The largest spectrum utilization ratio, among the four algorithms, with a dedicated backup path protection was obtained for BB_Cost and BB_Length, with a slight advantage for BB_Cost.

The smallest network utilization ratio was obtained for the MCMF algorithm. However, it should be remembered that the MCMF algorithm determines the working and dedicated backup paths for which the indexes of FSUs are the same. This leads to a large defragmentation of the spectrum, increasing the number of rejected requests and the bandwidth blocking probability, which in turn leads to a reduction in the spectrum utilization ratio in the network. The smallest bandwidth utilization ratio was obtained for the Primary FF Modified Backup LF algorithm; however, in this case, the spectrum on the backup paths is shared.

For each request, BB_Length determines the length sum of the paths, while BB_Cost determines the cost sum of the paths equal to, by definition, the product of the length and the required number of FSUs for each path. Thus, it can be assumed that BB_Cost determines the BB_Length solution in which each path is weighted by the required number of FSUs. This justifies the high convergence of all the results obtained from BB_Cost and BB_Length, which are shown in Figure 4, Figure 5, Figure 6, Figure 7, Figure 8 and Figure 9.

**Figure 10:**
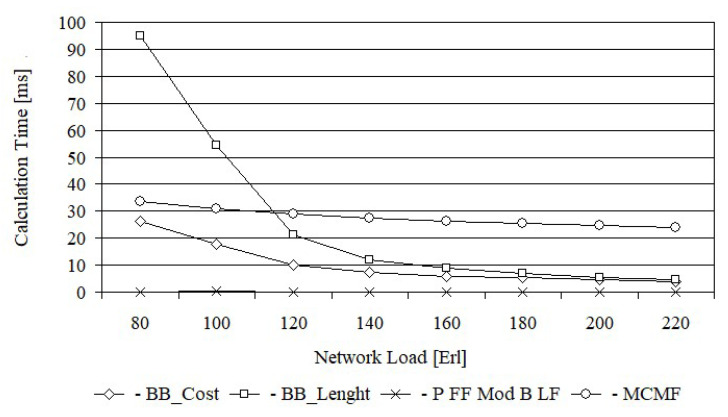
The average calculation time for Euro 28.

**Figure 11:**
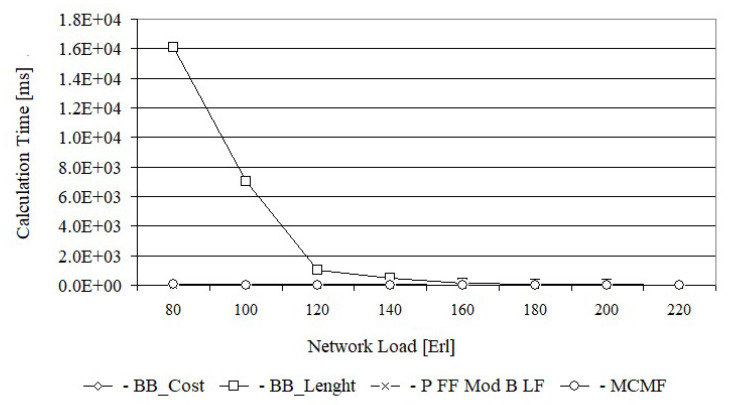
The average calculation time for US 26.

Figure 10 shows the calculation time of each of the tested algorithms for the Euro 28 network, while Figure 11 shows for US 26 networks. This time has been averaged after all simulation runs for each load. Path calculation time was taken into account when the request was successful or blocked. From Figure 10, it follows that the average calculation time of paths for the BB_Cost and BB_Length algorithms, depending on the network load, is exponential. This is due to the fact that the number of visited nodes in the generated solution tree, related to the number of analyzed paths, is exponential. Hence, the calculation time associated with the analysis of these nodes is also exponential.

It should be noted, however, that the calculation time of paths for the BB_Length algorithm is definitely larger for the same load than for the BB_Cost algorithm. For example, for a load of 80 Erl., the average calculation time for the BB_Length algorithm is equal to 95.0 ms, while for BB_Cost it is equal to only 26.4 ms. This difference is due to the fact that the lower bound of BB_Cost is more effective than lower bound of BB_Length, thus allowing a much larger number of nodes in the generated solution tree to be closed.

The path calculation time for the MCMF algorithm is a decreasing function of the network load and for small load (80 Erl.) is comparable to the time obtained for BB_Cost algorithm. The path calculation time for the Primary FF Modified Backup LF algorithm is properly independent of the network load and is equal to 0.18 ms.

From Figure 11, we can draw similar conclusions, however in this case the calculation times of the BB_Cost, MCMF and FF Modified B LF algorithms are dominated by the calculation time of the BB_Length algorithm, which for the load of 80 Erl is equal to 16 s. Such a long calculation time of path made it impossible to determine the characteristics for a lower load of the network.

A queue was used to search the generated solution tree. A much better structure for the generated solution tree would be the structure of the red-black tree, which is characterized by low computational complexity of operations such as inserting or removing nodes from the tree. The use of such a structure would certainly shorten the simulation time. However, it is known that BB_Cost and BB_Length are not scalable due to the exponential function of computational complexity and have been proposed to obtain exact solutions for different objective functions.

It should be noted that the determined pair of paths by BB_Cost or BB_Length for each request is optimal from the point of view of the adopted objective function, i.e., the occupied bandwidth or the length of the paths, respectively. In the case of the MCMF algorithm, which determines FSUs with the same indices on both paths, the objective function is the path length and the solution is heuristic. Assuming the same objective functions, one can speak of a trade-off between optimality (BB_Length) and feasibility (MCMF). However, when comparing other characteristics, it is no longer possible to speak of optimality because optimisation of bandwidth or path length does not necessarily lead to optimisation of the number of rejected requests, bandwidth blocking probability and utilisation of the network.

## 7. Summary and Conclusions

The online RSA problem with DPP is considered in this article. The solution to this problem is to determine, for the request, a pair of link-disjoint paths: working and dedicated backup path that meet the spectrum continuity and contiguity constraints, and non-overlapping spectrum constraints for adjacent connections on both paths. The considered problem also includes the optimization of the modulation level on each path.

Three algorithms have been proposed to solve this problem. The first of them is a heuristic algorithm, which calculates the solution assuming that indexes of the spectrum units on the working and backup path are the same. This assumption forces the use of the same units on the working path, which is generally shorter than the backup path. This assumption, on the one hand, causes spectrum waste and increases bandwidth blocking probability, but on the other hand it allows a simple, inexpensive and fast way to connect customers to the network. This should be important for network operators in the implementation phase of elastic optical networks.

The next two algorithms determine exact solutions. The first one, BB_Cost, calculates the paths with the smallest total cost, while the second one, BB_Length, calculates paths with a minimum total length. These algorithms enable the verification of the MCMF algorithm with the adopted assumption and give the answer to the question whether to optimize the total cost of paths or the total length of paths in order to minimize the bandwidth blocking probability.

The last Primary FF Modified Backup LF algorithm is heuristics that allow to specify reference results for the obtained results. This algorithm calculates working and shared backup path, from sets of k-shortest paths.

The conducted research, based on two example networks, EU 28 and US 26, showed that the proposed MCMF algorithm can be used in flexible optical networks, but with a small network load (in the considered examples, up to 80 Erl.). In addition, it has been shown for two exemplary networks that the optimization of the total cost of paths provides a slightly lower bandwidth blocking probability than the optimization of the total length of paths.

The MCMF algorithm computes the working and protection paths with the required number of FSUs with the same indices. Both the different lengths of the two paths and the same indexes of FSUs, attractive from a practical point of view, are hard to optimize. Therefore, it was known in advance that basic characteristics such as number of rejected requests, bandwidth blocking probability and utilization of the network would be worse than the characteristics obtained from the BB_Length and BB_Cost algorithms. It was also expected that the characteristics determined from the Primary First-Fit Modified Backup Last-Fit algorithm would be much better than those obtained from the other algorithms, since it is an algorithm for shared backup path protection.

The idea of this paper is to show the discrepancy between the obtained solutions based on BB_Length and BB_Cost and the solution worthy of attention from a practical point of view. Moreover, in MCMF, it was assumed that all requests will have the required number of slots with the same indices on both paths. In real-world conditions, making such strict assumptions may apply to only a small fraction of requests.

Further research should lead to algorithms solving the online RSA problem with a dedicated path protection, assuming that only a small section of network customers require a simplified way of connecting to the network.

## Figures and Tables

**Table 1:** Modulation formats used and distance for each of them.

Modulat.	Level	Reach *l*	Bit-Rate of
Format	m	[km]	FSU [Gb/s]
BPSK	1	2000 <l	12.5
QPSK	2	1000 <l≤ 2000	25.0
8-QAM	3	500 <l≤ 1000	37.5
16-QAM	4	l≤ 500	50

## Data Availability

No new data were created or analysed in this study. Data sharing is not applicable to this article.
